# Current status of stem cell therapy for type 1 diabetes: a critique and a prospective consideration

**DOI:** 10.1186/s13287-024-03636-0

**Published:** 2024-01-29

**Authors:** Mohamed A. Ghoneim, Mahmoud M. Gabr, Sawsan M. El-Halawani, Ayman F. Refaie

**Affiliations:** The Urology and Nephrology Center, Mansoura, Egypt

**Keywords:** Diabetes, Stem cells, Insulin-producing cells, Transplantation, Exosomes

## Abstract

Over the past decade, there had been progress in the development of cell therapy for insulin-dependent diabetes. Nevertheless, important hurdles that need to be overcome still remain. Protocols for the differentiation of pluripotent stem cells into pancreatic progenitors or fully differentiated β-cells have been developed. The resulting insulin-producing cells can control chemically induced diabetes in rodents and were the subject of several clinical trials. However, these cells are immunogenic and possibly teratogenic for their transplantation, and an immunoisolation device and/or immunosuppression is needed. A growing number of studies have utilized genetic manipulations to produce immune evasive cells. Evidence must be provided that in addition to the expected benefit, gene manipulations should not lead to any unforeseen complications. Mesenchymal stem/stromal cells (MSCs) can provide a viable alternative. MSCs are widely available from many tissues. They can form insulin-producing cells by directed differentiation. Experimentally, evidence has shown that the transplantation of allogenic insulin-producing cells derived from MSCs is associated with a muted allogeneic response that does not interfere with their functionality. This can be explained by the immunomodulatory functions of the MSC subpopulation that did not differentiate into insulin-producing cells. Recently, exosomes derived from naive MSCs have been used in the experimental domain to treat diabetes in rodents with varying degrees of success. Several mechanisms for their beneficial functions were proposed including a reduction in insulin resistance, the promotion of autophagy, and an increase in the T regulatory population. However, euglycemia was not achieved in any of these experiments. We suggest that exosomes derived from β-cells or insulin-producing cells (educated) can provide a better therapeutic effect than those derived from undifferentiated cells.

## Introduction

Diabetes mellitus (DM) is a metabolic disease that is a major health concern. It results from a deficiency of insulin production in type 1 diabetes mellitus (T1DM) or an inability to utilize this hormone as occurs in type 2 diabetes (T2D). Globally, more than 400 million people suffered from DM in 2014 compared to 108 million in 1980. If this trend continues the number is expected to increase to more than 600 million by 2045 [1]. Of these, T1DM accounts for approximately 10% of cases. T1DM pathogenesis involves autoimmune-mediated destruction of insulin-producing β-cells in pancreatic islets. Evidence supporting the autoimmune basis of T1DM development includes the presence of lymphocytic infiltrate around and in islets and the appearance of autoantibodies against multiple islet autoantigens. As the β-cell mass declines, insulin secretion decreases until the available insulin is inadequate to maintain normal blood glucose levels. Administration of exogenous insulin is the main treatment for T1DM patients. While the maintenance of appropriate glycemic control is possible with insulin therapy, it fails to prevent microvascular complications in many subjects. Furthermore, inaccurate insulin delivery results in lack of glycemic control and/or hypoglycemia. Islet transplantation can provide an effective treatment for patients with type T1DM [2]. Despite promising outcomes, the essential problems with islet transplantation are the need for immunosuppression and the scarce donor supply. Alternatively, stem cell-derived insulin-producing cells can provide an unlimited supply. To this end, the use of embryonic, neonatal, induced pluripotent and mesenchymal/stromal cells has been reported and was the subject of several systematic reviews [3–5]. In this contribution, critical appraisal has been limited to the key experimental findings and relevant clinical trials. The objective is to identify the limitations that have to be overcome before stem cell therapy for diabetes becomes reliable and reproducible. As an alternative to cell therapy, the use of stem cell-derived extracellular vesicles (EVs) is discussed as a future possibility.

## Pluripotent stem cells

Human pluripotent stem cells (hPSCs) include embryonic stem cells (hESCs) that are derived from the inner cell mass of the embryo and human induced pluripotent stem cells (hiPSCs) that are generated by somatic cell reprogramming. hESCs show unlimited replicative properties and have the potential to differentiate into any adult cell type. hiPSCs have the same ability to expand and differentiate as ESCs. However, the use of hiPSCs has fewer ethical considerations than hESCs. Moreover, hiPSCs provide an opportunity to use autologous cells, and hence, allogenic immune responses can be avoided [6]. Some differences between hESCs and hiPSCs have been reported in some studies. These include gene expression profiles, epigenetic modifications such as DNA methylation and genetic stability [7, 8].

### Differentiation of hPSCs into pancreatic β-cells

In a key study, Kubo and associates reported the development of definitive endoderm from embryonic stem cells (ESCs) in culture using activin A [9]. Based on this finding, D'Amour et al. [10] developed a protocol for the differentiation of hESCs into pancreatic progenitor endoderm cells (PECs). The authors maintain that after transplantation, these cells undergo further maturation under the influence of the in vivo environment [11]. Using a four-step protocol, Rezania et al. reported the efficient differentiation of hESCs in vitro into pancreatic progenitors which further develop in vivo into mature pancreatic endocrine cells [12]. Two years later, the same group of investigators reported the successful differentiation of hESCs into insulin-producing cells (IPCs) using a seven-step protocol that mimics embryologic development [13]. At stage 4, pancreatic progenitors were obtained, while at stage 7, mature β-cells were produced. A comparison between these 2 differentiation products was outlined by Memon and Abdelalim [14]. In summary, in vitro differentiation of hPSCs to pancreatic progenitors takes 2 weeks while their differentiation to insulin-secreting β cells is longer and takes more than a month. As a result, the detection of human C-peptide secretion occurs 3–4 months after transplantation of pancreatic progenitors and approximately 2 weeks after transplantation of fully differentiated β cells. Following their further differentiation in vivo, pancreatic progenitors yield islet-like formations expressing insulin, glucagon, and somatostatin. With transplantation of β cells, insulin is only produced.

Further optimizations improved the differentiation efficiency of β-cell generation from 33% [15] to 75% [16]. A clear pathophysiologic breakdown of the 7 differentiation stages was recently outlined by Verhoeff et al. [17]. For each stage, the biochemical pathways, the cell markers and the required duration were identified. Furthermore, the authors suggested an optimized protocol for selective teratoma elimination.

### Clinical transplantation of hPSC differentiation products

Transplantation of IPCs derived from hESCs has 2 major challenges: immunogenicity and teratogenicity. Consequently, the transplantation of these cells requires their enclosure within an immunoisolation device and/or the use of immunosuppressive agents. To this end, an immunoprotective device was developed by ViaCyte [ViaCyte Inc. San Diego, CA, USA]. The device was loaded with PECs derived from hESCs. The data showed that subcutaneous transplantation of this combination [VC-01TM] controlled chemically induced diabetes in rodents [18]. Henry et al. [19] and Odorico et al. [20] reported the results of the first clinical trial using this system for islet replacement in patients with T1DM. The study was an open-label trial that included 19 patients. A high degree of variability in outcomes was observed presumably due to poor vascularization and/or foreign body response to the device components. The 12-week-old explants showed minimal cell survival due to hypoxia. The trial was paused to improve the configuration of the encapsulation device. In a more recent trial, PECs were loaded into a modified device with wide pores to allow vasculature to grow into its lumen [VC-02]. The aim was to enhance cell survival through better oxygenation and improve metabolic exchange through this open system. However, this device does not offer immune protection. Immunosuppressive agents must be administered to prevent an allogeneic immune response. The early clinical outcomes of an open-label trial using this modified device were recently reported by two groups of investigators. The results of 17 subjects with type 1 diabetes were reported by Shapiro and associates [21]. Six patients demonstrated a stimulated C-peptide response ≃ 6 months *post-transplantation* following a mixed-meal tolerance test. At 1 year, subjects without a measurable C-peptide response were withdrawn from the study and their devices were explanted. Explants were dominated by α cells, and only a subset demonstrated a mature β-cell phenotype. The authors noted that despite the presence of a measurable C-peptide among some patients in this pilot study, a significant clinical benefit was not observed. In the second study, Ramzy et al. [22] disclosed their results from 15 patients using the same devices. These authors reported that the total daily insulin requirements were reduced. Only one patient had an ≃ 50% reduction in his insulin needs 1 year post-transplantation. Although both studies provided a proof of concept that stem cell-derived endoderm cells can be engrafted in patients with T1DM and differentiate into islet cells, insulin independence was not reported in any of their patients. The lack of a complete therapeutic response was attributed to an insufficient number of engrafted cells and pericapsular fibrous tissue deposition. Further optimization of the implantation device is needed.

Fully differentiated IPCs were developed from allogeneic pluripotent stem cells by Vertex Pharmaceutical Incorporated (Boston, Mass, USA). In phase I/II, open-label clinical trial (NCT04786262), two type T1DM patients with impaired hypoglycemic awareness were enrolled. IPCs were transplanted into the portal system. Immunosuppressive agents also had to be given. Early impressive data from the first patient were announced by Vertex. On day 270, the patient achieved insulin independence with a HbA1c level of 5.2%. The results from the second patient were not as good. By day 150, the exogenous insulin requirements decreased by 30% only and the HbA1c became 7.1%. However, results have not yet been published. Currently, Vertex is planning to enroll an additional 17 patients. The downside of such treatment is the imperative immunosuppression requirement. In effect, there is a trade-off between the frequent administration of insulin for the use of immunosuppressive agents.

### Immunomodulation of hPSC-derived IPCs

To circumvent the adverse effects of immunosuppression, a growing number of studies have utilized immune and genome engineering [23–25]. Sintov et al. [26] used 2 approaches to identify the genes that are responsible for stem cell–islet immunogenicity: single-cell RNA sequencing to characterize responses to immune challenge and CRISPR genome screening to assess the underlying source of these responses. The strongest effect was the upregulation of interferon-stimulated gene expression. The secreted interferon leads to an inflammatory cascade with activation of the JAK/STAT pathway. It was suggested that a practical and promising approach is to specifically target the downstream components of the JAK/STAT pathway by the depletion of chemokine ligand 10 [CXCL10]. The authors reported that peripheral blood mononuclear cells (PBMCs) preloaded with cell trace violet to measure proliferation rates showed reduced T-cell proliferation when cocultured with CXCL10-depleted stem cell-derived islets. In addition, these cells evaded an alloimmune attack when transplanted into humanized mice. The same group of investigators used a different approach to generate immunoinvasive human stem cell-derived islets [27]. They observed that targeting human HLAs and PD-LI does not sufficiently protect these cells from allogeneic immune responses and suggested that the addition of knock-in genes generated a local tolerogenic environment. The researchers further genetically engineered their cells to secrete IL-10, TGFβ, and modified IL-2. Transplantation of these cells treated diabetes in NOD mice for up to 8 weeks. Wang and associates [28] also genetically engineered MSCs-derived from mice to express PD-LI and CTLA4-Ig. These modified MSCs were used as accessory cells for islet transplantation. The authors reported that with this protocol there was an improvement in the outcomes of both syngeneic and allogeneic islet transplantation in diabetic mice, which resulted in allograft survival for 100 days without immunosuppression. An open-label first-in-human trial (NCT05210530) to evaluate the safety and tolerability of a device developed by CRISPR Therapeutics (Boston, MA, USA) and Vacate was initiated. The product (YCTX210A) has two components: genetically modified allogeneic pancreatic endoderm cells that induce immune evasiveness and a perforated device that delivers and retains the cells. Treatment of the first patient with this system was announced in February 2022. No results have been posted yet. Genetic modification of cells can have potential risks. The transplantation of immune evasive cells with a tumorigenic potential can also have adverse results. Evidence must be provided that in addition to the expected benefits, genetic manipulations must not cause any unforeseen complications.

## Mesenchymal stem/stromal cells (MSCs)

### General functions

MSCs are widely available from many tissues and can be readily expanded in vitro. Naive MSCs can home to injured tissues thereby expediting their repair [29]. These cells are immune evasive due to the lack of expression of HLA class II antigens and the costimulatory molecules CD40, CD80, and CD86. Furthermore, MSCs exert an immunomodulatory function by releasing soluble factors as well as via cell-to-cell contact when activated by proinflammatory cytokines [30, 31]. It was assumed that upon in vivo administration, MSCs would repair damaged tissue by engraftment and subsequent differentiation. However, many studies have provided evidence that MSCs are not easily engrafted into target tissues and that transplanted cells eventually die or are destroyed [32]. As a result, the paradigm of MSC-mediated function has shifted toward secretom-based signaling rather than cellular engraftment and differentiation. However, this concept was recently challenged by Boregowda and associates [33], who proposed that MSCs exert their functions by their stem/progenitor nature as well as by their paracrine effects. Phinny et al. proposed that while stem/progenitor functions promote cell repair, angiogenesis and paracrine functions are anti-inflammatory and immunomodulatory [34]. Furthermore, these authors demonstrated that *TWIST1* expression levels can predict the therapeutic function of a given MSC population. A high level of *TWIST1* mRNA expression is associated with stem/progenitor functions while a low expression level with their paracrine immunomodulatory properties. As a result, it would be possible to select the specific MSC lines that can serve the required function. Recently, Van Grouw and associates have developed a predictive machine learning model to identify the potency of the MSC immunomodulation [35]. They identified the extracellular metabolites generated throughout their expansion by nuclear magnetic resonance and the resulting intracellular metabolites by mass spectrometry. They reported that the presence of the extracellular metabolites proline, phenylalanine, and pyruvate and the intracellular metabolites such as sphingomyelins can be used to identify MSC lines with high immunomodulatory potency.

### Undifferentiated MSCs and diabetes mellitus

 There are several mechanisms by which MSCs can exert their therapeutic benefit. They can home to the site of injury and support the repair of pancreatic islets [36, 37]. MSCs also enhance islet graft vascularization [38]. Through their immunomodulatory function, they can provide a therapeutic means for early-onset T1DM [39]. Motivated by these promising properties, the use of MSCs for the treatment of DM was evaluated in several clinical trials. Hu et al. randomized 29 patients as follows: 15 received Wharton's Jelly-derived MSCs intravenously and 14 received the conventional treatment and served as controls [40]. The authors reported an increase in the mean C-peptide levels and a decrease in insulin requirements in MSC-treated patients. Carlsson and associates used autologous bone marrow-derived MSCs in 10 patients [41]. The investigators reported an increase in the C-peptide response in a mixed-meal tolerance test at 1 year of follow-up. In a trial by Araujo and colleagues, 13 patients were randomized as follows: 8 received allogenic AT-derived MSCs and vitamin D and 5 served as controls [42]. Three months after intervention, the MSC-treated group had lower insulin requirements and HbA1c levels. Izadi et al. treated 11 patients with autologous bone marrow-derived MSCs [43]. They reported that MSC treatment in early diagnosed T1DM improved the HbA1c and C-peptide levels. It was also observed that there was a shift from proinflammatory cytokines to anti-inflammatory ones. Carlsson et al. used umbilical cord-derived MSCs to treat 10 patients with early-onset T1DM. They reported that the insulin requirements were not changed in the treatment group while it increased by a median of 10 units/day in the control group [44]. He and associates recently reported the results of a thorough systematic review and meta-analysis of randomized controlled trials that tested the clinical efficacy and safety of MSCs in diabetic patients [45]. These authors identified 176 full-text papers. However, 169 studies were excluded because they involved nonhuman experiments or were not randomized and controlled. It was reported that MSC infusion improved the HbA1c levels, although there were no significant differences in the fasting glucose or C-peptide levels. Although these studies demonstrated a potential benefit of systemic MSC administration for the treatment of early-onset T1DM, considerable number of uncertainties remain and a final conclusion cannot be drawn. The source of MSCs was heterogeneous, the dosage of cells variable and the number of patients limited [46]. In view of these limitations, a number of high-quality, large-scale randomized studies are needed to provide a definitive conclusion [47].

To capitalize on their immunomodulatory function, MSCs were cotransplanted with pancreatic islets in some recent reports. Wang et al. harvested bone marrow-derived MSCs from three chronic pancreatitis patients who were scheduled to receive islet autotransplantation [48]. The islets were cotransplanted with MSCs via the portal vein. The authors reported that the procedure was safe. The patients had lower 12-month fasting glucose levels and a better quality of life compared with controls. Ishida and associates pretreated MSCs with inflammatory cytokines to enhance their immunomodulatory properties [49]. They cotransplanted these preactivated MSCs with pancreatic islets into the portal vein. The authors reported a remarkable improvement in graft survival with preconditioned MSCs but not naive MSCs.

### Differentiated MSCs and diabetes

On a different note, evidence has shown that under certain culture conditions, MSCs can form cells that do not belong to a mesodermal lineage [50, 51]. As early as 2004, the successful differentiation of murine bone marrow-derived MSCs (BM-MSCs) into insulin-producing cells was reported by three investigators [52–54]. These early observations were reproduced by using adipose tissue-derived MSCs (AT-MSCs) [55, 56]. These early findings were challenged [57–59]. It was suggested that insulin from the culture media could be absorbed by and sequestered in these cells [60]. Gabr et al. [61] provided a final proof of concept. These investigators differentiated human BM-MSCs into IPCs. In their study, all the required criteria for the successful production of IPCs were met [62]. These findings were later confirmed by several studies [63–66]. While bone marrow and adipose tissue are the most studied, other sources for the differentiation of MSCs into *IPCs* were also reported. These include umbilical cord [67] amniotic fluid [68], Wharton’s jelly [69], and dental pulp [70]. Stored umbilical cord-derived MSCs can serve as an autologous source for their donors in case of future need. Recently, there has been increasing interest in the use of Wharton's jelly-derived MSCs to generate IPCs [71]. These cells have a high replication capacity without observed senescence for up to 80 population doublings [72].

The reported results of allogenic immune responses following the transplantation of MSC-derived IPCs are controversial [30]. Transplantation of IPCs derived from human MSCs into humanized mice can provide evidence. Such an experiment was carried out in our laboratory, and the results were recently published [73]. Allogeneic IPCs derived from hAT-MSCs were transplanted into STZ-diabetic humanized mice (NOG-EXL mice, Taconic, Bioscience, Rensselaer, NY, USA). Collectively, the results of this study confirmed that the transplantation of allogeneic hAT-MSCs into diabetic humanized mice normalized their blood sugar levels. An allogeneic immune response was not detected. Differentiated IPCs accounted for only ~ 20% of the transplanted cells. It can be hypothesized that the undifferentiated population exerted an immunomodulatory effect. This suggestion is in line with the findings of Wu et al. [74], who reported that third-party undifferentiated MSCs improved the outcomes of islet transplantation in humanized diabetic mice. The results of this study suggest the potential implementation of cell therapy without immunosuppression, encapsulation, or genetic manipulations for insulin-dependent diabetic patients. However, to our knowledge, clinical trials using MSC-derived IPCs for the treatment of diabetes have not been reported.

## Challenges and limitations of stem cell therapy for T1DM

IPCs derived from pluripotent stem cells face two important problems: allogeneic immune responses and their potential for tumorigenesis. Encapsulation within an immunoisolation device or the use of immunosuppression is necessary. Encapsulation has inherent drawbacks. Further refinements of the currently available encapsulation systems are required. Cell hypoxia must be avoided, and adequate vascularization ensured. The induced pericapsular fibrosis resulting from a foreign body tissue reaction must also be minimized. If these issues are not resolved, satisfactory and meaningful results of cell transplantation within an encapsulation device will be difficult to achieve. The lifelong need for immunosuppression has serious adverse side effects. Moreover, some utilized immunosuppressive agents are diabetogenic.

For clinical applications, the site of implantation of stem cell-derived IPCs imposes an additional challenge. There are three commonly reported sites: the portal system, the omentum, and the subcutaneous tissue. The portal vein remains the preferred site for islet transplantation. However, due to hypoxia and blood-mediated inflammatory reactions, a substantial proportion of islets are destroyed following infusion into the portal vein [75]. However, in a recent study, it was reported that for islet transplantation, intraportal infusion provides superior engraftment and function compared to extrahepatic transplantation [76]. The omentum has a rich blood supply and a large surface area that allows the hosting of a large number of cells. Successful transplantation into an omental pouch was reported by two groups of investigators [77, 78]. Nevertheless, laparoscopic intervention was required. The subcutaneous site is easily accessible, and a minimally invasive procedure is needed. The main disadvantage of this site is its poor blood supply. Yu et al. reported successful islet transplantation under the skin when cells were admixed with what was called an islet viability matrix (IVM) [79]. If the results of this study can be reproduced using MSC-derived IPCs, an important step in the application of cell therapy for diabetes. It remains to be determined whether cells transplanted within such a matrix have an accessible and adequate early oxygen supply. A summary of stem cell-derived IPCs is given in Table 1.

**Table 1:** Different sources for human stem cell-derived insulin-producing cells

Cell of origin	Differentiation protocol	Time required for differentiation (days)	Differentiation product	Clinical trial	Site of transplantation	Encapsulation	Immunosuppression
Embryonic stem cells	[11, 12]	14	Pancreatic progenitor	Yes [19] NTC: 02239354	Subcutaneous	Yes (VC-01)	No
Pluripotent stem cells	[13, 15–17]	14	Pancreatic progenitor	Yes [21, 22]	Subcutaneous	Yes (VC-02)	Yes
Pluripotent stem cells	[8, 98]	30–36	Insulin-producing cells (VX-880)	Yes*	Intraportal	No	Yes
Mesenchymal stromal/stem cells	[61, 63–66]	10–21	Insulin-producing cells	No only experimentally in rodents [61, 65]	Under the renal capsule	No	No

*Announcement by vertex pharmaceutical

Finally, regardless of the source of stem cell-derived IPCs, additional questions must be addressed. The number of cells that are required to achieve insulin independence has to be quantified, and their functional longevity should also be determined.

## Prospects: MSC-derived exosomes?

Extracellular vesicles (EVs) are membrane vesicles that are naturally released from cells including MSCs. They are enclosed within a lipid bilayer and cannot replicate as they do not contain a functional nucleus. EVs in the size range of 30–100 nm are known as exosomes, a term that was coined by Trams et al. [80]. Exosomes are the only class of EVs, known to be derived from endosomes through invagination of the endosomal membrane to form multivesicular bodies (MVBs). Exosomes are released by the fusion of the MVBs with the plasma membrane. Exosomes are known to function as a one-way conveyers of cellular material from secreting cells to recipient's cells to modulate their functions. This can be achieved by contact-dependent signaling [juxtracrine signaling], by the release of exosomes in close proximity to the source [paracrine signaling], or by endocrine signaling whereby exosomes are then transported systemically via the bloodstream to act distally. In a seminal experiment, Valadi et al. [81] cocultured exosomes derived from a mouse mast cell line [Mc/g] with a human mast cell line (HMC-1). Thereafter, three distinct mouse proteins were identified in human cells. Since exosome-derived RNA from mast cells could be transferred to other mast cells but not to CD4+ cells, it was concluded that exosomes modulate recipient cells through specific receptor–ligand interactions. The authors maintained that the transfer of exosome-derived miRNAs or mRNAs to the recipient cells allows for gene-based communication between mammalian cells resulting in the modulation of protein secretion by recipient cells. This concept was later supported by reports from several investigators [82–85]. It was also shown that MSCs can produce larger amounts of exosomes than other cells [86]. The resulting exosomes have functions similar to MSCs, such as the promotion of cell repair and the modulation of immune responses. As a result, MSC-derived exosomes have been tested as a cell-free therapy for the treatment of a variety of pathologic conditions [87].

## MSC-derived exosomes and diabetes mellitus

As an alternative to cell therapy, the potential role of MSC-derived exosomes in the treatment of DM has been explored in several experimental studies. In an in vitro study, Favaro et al. [88] cocultured hBM-MSCs or their derived EVs with dendritic cells (DCs) obtained from type 1 diabetic patients. The researchers reported that MSCs or their derived EVs induced the formation of IL-10-secreting regulatory DCs. They suggested that these regulatory DCs inhibit inflammatory T-cell responses to islet antigens and can prevent the progression of T1DM. Shigemoto-Kuroda et al. reported that hMSC-derived EVs could effectively prevent the onset of autoimmune diabetes in NOD/*SCID* mice [89]. They demonstrated that these EVs inhibited the activation of antigen-presenting cells and suppressed the development of proinflammatory T cells. Nojehdehi and colleagues showed that exosomes derived from the AT-MSCs of C57BL/6 mice exerted an immunomodulatory effect when administered to STZ-induced diabetic mice in a model of the same strain [90]. They attributed this function to an increase in the T regulatory cell population. Sun et al. established a rat model of T2DM using a high-fat diet followed by STZ administration [91]. Exosomes were prepared from the cell culture supernatant of human umbilical cord MSCs and injected into the tail vein of diabetic Sprague Dawley rats at a dose of 10 mg/kg every 3 days for 5 cycles. Compared to controls, treatment with exosomes reduced blood sugar levels, enhanced insulin sensitivity, and promoted GLUT4 expression in skeletal muscles and glycogen storage in the liver. In another experiment, He et al. prepared exosomes from human umbilical cord stem cells [92]. They established a T2DM model in rats by using a high-fat diet followed by STZ administration. Exosomes were injected intravenously via the tail vein at a dose of 10 mg/kg in 200 μl of PBS every 3 days for 2 months. There was a significant reduction in blood glucose levels following the administration of exosomes or the engraftment of human MSCs [5 × 10^6^/rat]. The intraperitoneal glucose tolerance test (IPGTT) and intraperitoneal insulin tolerance test (ITT) revealed that exosome administration improved glucose metabolism and increased insulin sensitivity. Furthermore, autophagy-related proteins were increased following treatment with exosomes. The authors suggested that exosomes improved glucose and lipid metabolism by promoting autophagy. Yap et al. prepared EVs derived from human umbilical cord MSCs [93]. These EVs were used in two experimental settings: in vitro and in vivo. In the in vitro study, the administration of 20 μg/ml of EVs resulted in an 80–90% increase in glucose uptake in human skeletal muscles. In the in vivo study, diabetes was chemically induced in rats. The diabetic rats received EVs by I.V. at a dose of 1 mg/kg body weight every 3 days for a total of 5 cycles. Treatment with EVs improved glucose tolerance, HbA1c, and insulin sensitivity. Exosomes were isolated from the conditioned medium of an insulinoma cell line [MIN6] by Sun and associates [94]. The administration of these exosomes into STZ-induced diabetic mice improved glucose tolerance, increased the insulin content, and preserved the architecture of pancreatic islets. Guo and associates cocultured exosomes derived from a mouse insulinoma cell line (MIN6) with human iPSCs for 21 days [95]. After 7 days of coculture, the proportion of insulin-positive cells by flow cytometry was 22.3% in the exosome-treated cell population, while it was only 11.9% among the controls. The treated iPSCs showed higher expression of the relevant pancreatic endocrine genes. Furthermore, miR-706, miR-709, miR-466-c-5p, and miR-423-5p expression was upregulated in the exosome-induced iPSCs. Diabetes was then chemically induced in C57BL/6 mice. The diabetic animals were divided into the following 3 treatment groups: those that received iPSCs [1 × 10^7^/mouse], those that received exosome-induced iPSCs, and those that received siAgo2 (Argonaute 2) exosome-induced iPSCs to reduce miRNA synthesis. Cells from the three groups were engrafted under the renal capsule of diabetic mice. There was a 50% reduction in blood glucose concentration among the exosome-induced iPSCs compared to that of the other two groups. There was also a decrease in β cell-specific genes among the siAgo2-treated exosomes. It was concluded that exosomes induce iPSC differentiation in vitro and that this process is mediated by exosomal miRNAs. To determine the optimal route for administration, human umbilical cord MSC-derived EVs were tagged with iodine-124 [96]. The labeled EVs were injected either I.V. in the tail vein or intra-arterially via the coeliac artery of nondiabetic Lewis rats. PET imaging revealed that the predominant uptake of the administered exosomes occurred in the liver following either injection routes.

It is worth mentioning that the used protocols were not uniform and the suggested possible therapeutic benefit were variable. Furthermore, in all these experiments, there was a reduction in blood glucose levels among the treated diabetic animals, but euglycemia was not achieved. It is likely that the exosomes used were derived from undifferentiated MSCs and not from mature β cells or IPCs. It is reasonable to assume that exosomes derived from β cells or human IPCs (educated exosomes) may achieve better therapeutic results. An experimental study by Bai et al. supports this hypothesis [97]. These investigators utilized a differentiation protocol based on previously reported methods by Pagliuca et al. [98] and Millman et al. [8]. At the final stage, the differentiated iPSCs were incubated with 15 μg/ml of EVs derived from 1 × 10^5^ human β cells. The EVs were replaced every 3 days for 15 days. The authors reported that this coculture promoted the differentiation of iPSCs to what they called i-β cells. In vitro, these i-β cells exhibited the functional properties of pancreatic β cells. There was a significant upregulation of insulin which was reversed in the presence of miR-212 and miR-132 inhibitors. These miRs enhance the differentiation of β cells by stabilizing NGN3 expression [99]. In vivo, 2 × 10^7^ i-β cells were transplanted under the renal capsule of STZ-induced diabetic SCID mice. Following transplantation, the blood glucose levels were normalized at all time points during a follow-up period of 4 weeks. However, the authors did not determine or quantify the extent of functional improvement following coculture of their differentiated iPSCs with β cell-derived EVs.

## EVs as an alternative to cell therapy

Preclinical studies of EVs utilized in the diagnosis and treatment of diabetes or its complications have been the subject of several reviews [100–104]. Only one clinical trial was posted in the ClinicalTrials.gov., in 2014 (NCT0213833). Since then, no results have been announced or reported. Meanwhile, experiences with stem cell-based therapy are common and are being continuously optimized [105]. However, the use of exosomes can avoid several problems associated with cell therapy notably necrosis and immune rejection. With exosomes, there is no possibility of teratogenesis since they have no nuclei. EVs can be stored and used as an off-the-shelf therapeutic tool that can be delivered on a timely basis [106]. Exosomes are administered intravenously, and their application can be repeated without an invasive procedure. *Furthermore*, they have an autonomous targeting ability and can be designed as carriers for specific molecules. Nevertheless, the therapeutic use of EVs is challenging because of the lack of standardized methods for their preparation or characterization. Moreover, the MSCs used as a source for exosome production are heterogeneous. As a result, the prepared samples may contain a mixture of EV subtypes. Witwer et al. [107] emphasized the need to combine the minimal criteria to identify MSCs as suggested by the International Society for Cell Therapy [108] and the recommendations of the International Society for Extracellular Vesicles, which were updated in 2018 [109]. In this manner, a framework can be defined for the preparation and characterization of EVs. Since global standardization of MSC-derived EVs has not yet been achieved, the final product should be defined by physical, biochemical, and functional attributes using reproducible assays.

## Concluding remarks

**Fig. 1:**
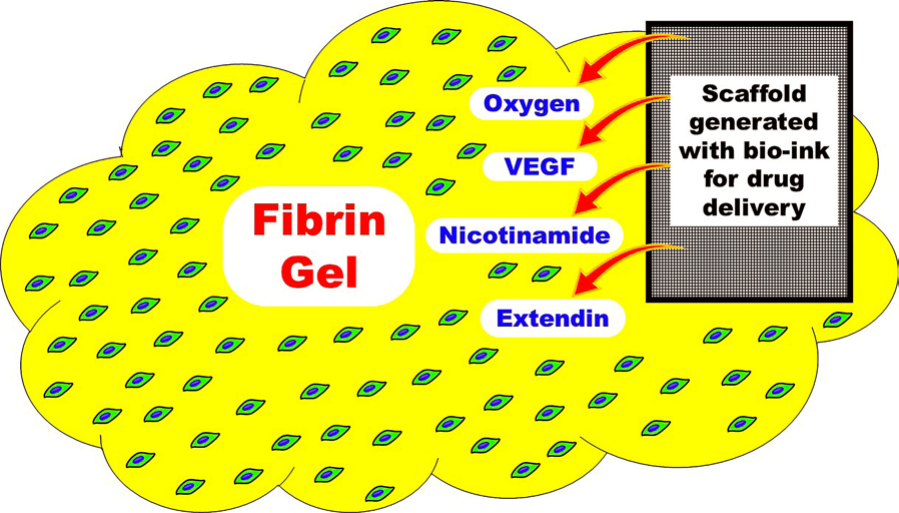
A suggested model for subcutaneous transplantation of MSC-derived IPCs. Cells are transplanted within a biological matrix. A small scaffold is cotransplanted for drug delivery. Oxygen for the immediate requirements, VEGF to induce early vascularization, nicotinamide to preserve islet viability, and exendin to promote further differentiation in vivo

Over the past decade, significant progress in stem cell therapy for diabetes has been achieved. Nevertheless, there are still several hurdles and challenges that have to be overcome before cell therapy can be reliably used for diabetes. The goal is to carry out successful cell transplantation without encapsulation, immunosuppression, or genetic manipulations. To achieve this goal, the use of MSC-derived IPCs provides a glimpse of hope. Compared to ESCs and iPSCs, MSCs have a negligible teratogenic risk [110]. Experimental evidence showed that allogenic transplantation of these cells does not evoke an immune response presumably based on the immunomodulatory functions of the undifferentiated population. The immunomodulatory function of MSC-derived IPCs can be further enhanced to overcome autoimmune reactions in individuals with T1DM [111–114]. The downside of MSC clinical applications is their heterogeneity which is dependent on the source of their procurement and the donor-to-donor variability [115, 116]. Attempts toward their standardization are currently under exploration [117]. The subcutaneous tissue would be an optimal site for cell transplantation [118]. However, the problem of poor vascularization has to be solved. A model for subcutaneous transplantation of MSC-derived IPCs is currently being tested in our laboratory. The cells are being transplanted within a fibrin matrix. In addition a small scaffold is being cotransplanted for drug delivery (Fig. 1).

**Fig. 2:**
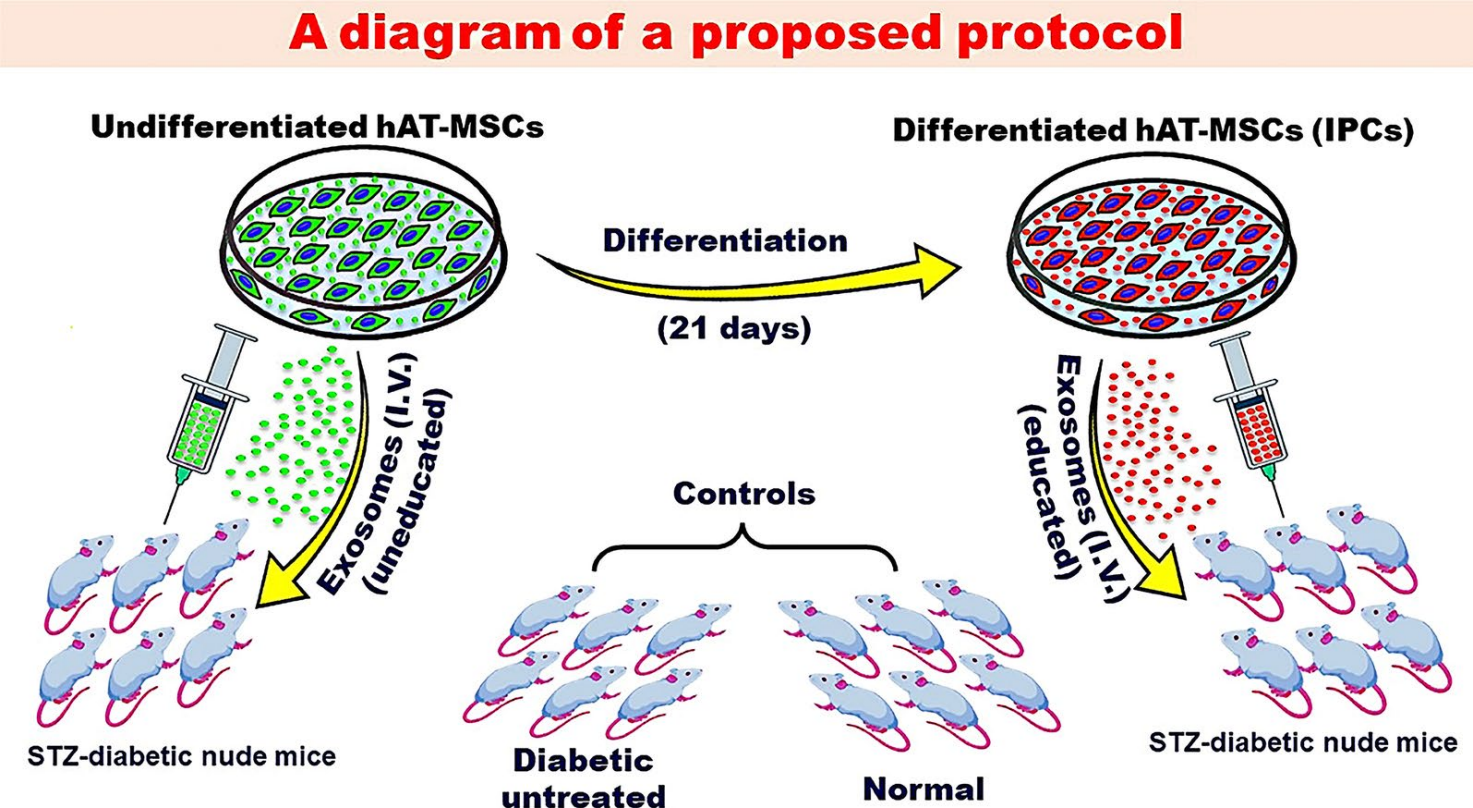
A suggested protocol to explore the potential benefits of MSC-derived exosomes. Exosomes are retrieved from the conditioned medium of naïve MSCs (uneducated exosomes) or differentiated IPCs (educated exosomes) and injected I.V. in STZ-diabetic mice. The results are compared to cell transplantation under the renal capsule

Exosomes are an exciting therapeutic option. We suggest that educated exosomes derived from IPCs may provide better therapeutic results than exosomes derived from undifferentiated MSCs. Experiments that explore this concept are underway in our laboratory (Fig. 2). Finally, it must be emphasized that cell or exosome therapy for insulin-dependent diabetic individuals can be meaningful and clinically justified only if the functional outcome is better than that of newly discovered medications or the ever-improving closed-loop insulin pumps.

## Data Availability

All materials are available.

## Abbreviations

MSCs: Mesenchymal stem cells
IPCs: Insulin-producing cells
DM: Diabetes mellitus
T1DM: Type 1 diabetes mellitus
ESCs: Embryonic stem cell
hESCs: Human embryonic stem cells
iPSCs: Induced pluripotent stem cells
PECs: Pancreatic progenitor endoderm cells
AT-MSCs: Adipose tissue-derived stem cells
BM-MSCs: Bone marrow-derived stem cells
PBMNs: Peripheral blood mononuclear cells
IVM: Islet viability matrix
EVs: Extracellular vesicles
MVBs: Multivesicular bodies
DCs: Dendritic cells
IPGGT: Intraportal glucose tolerance test
